# A 3D Digital Analysis of the Hard Palate Wound Healing after Free Gingival Graft Harvest: A Pilot Study in the Short Term

**DOI:** 10.3390/dj10060109

**Published:** 2022-06-13

**Authors:** Tiago Marques, Sara Ramos, Nuno Bernardo Malta dos Santos, Tiago Borges, Javier Montero, André Correia, Gustavo Vicentis de Oliveira Fernandes

**Affiliations:** 1 Faculty of Dental Medicine, Universidade Católica Portuguesa, 3504-505 Viseu, Portugal; tmmarques@ucp.pt (T.M.); spfframos@hotmail.com (S.R.); nbsantos@ucp.pt (N.B.M.d.S.); tborges@ucp.pt (T.B.); andrecorreia@ucp.pt (A.C.); 2 Center for Interdisciplinary Research in Health, 3504-505 Viseu, Portugal; 3 Department of Surgery, Faculty of Medicine, University of Salamanca, 37008 Salamanca, Spain; javimonteiro@usal.es; 4 Periodontics and Oral Medicine Department, University of Michigan School of Dentistry, Ann Arbor, MI 48109-1078, USA

**Keywords:** connective tissue, wound healing, plastic surgery, digital technology

## Abstract

Purpose: Within this context, this pilot study aimed to evaluate the healing dynamics process of the hard palate after free gingival graft harvesting in the short term (3 months), utilizing digital imaging technology and tridimensional analysis software. Furthermore, assessing the results found to verify the existence of a relationship between gender or age with tissue loss. Materials and Methods: For connective-tissue harvesting, fifteen patients with gingival recessions type (RT) 1 and RT2 were selected. On the surgery day (before the procedure) and after three months, palatal impressions were taken in all patients, and cast models were done for posterior model scanning. The following variables were analyzed: mean thickness alterations ($\bar{x}$ TA), maximum thickness loss (MTL), mean maximum thickness loss ($\bar{x}$ MTL), and volume alterations (VA). A descriptive and bivariate analysis of the data was done. The data were submitted for statistical evaluation and were significant if *p* < 0.05. Results: Fifteen patients were analyzed, 11 females (73.3%) and four males (26.7%). The patients’ average age was 28 ± 8.52 years (ranging between 16 and 48 years old). The palatal wound region’s mean thickness and volume changes were −0.26 mm (±0.31) and 46.99 mm^3^ (±47.47 mm^3^) at three months. There was no statistically significant result correlating age/gender with any variable evaluated. Conclusions: Connective tissue graft harvesting promoted changes with a standard volume and thickness loss of palatal soft tissue. A 3D digital evaluation was a non-invasive method with a reproducible technique for measuring thickness or volume after connective tissue is collected. There was no relationship between age/gender and any variables analyzed.

## 1. Introduction

Periodontal plastic surgery (PPS) aims to prevent and correct gingival and alveolar mucosa defects or the bone tissue caused by anatomical, traumatic, or disease-induced changes [1]. A significant portion of these surgeries is intended to cover recessions caused by the soft-tissue apical migration that exposes the root surface or implant [2]. They are also often conducted to increase the keratinized tissue width (KTW). The rationale behind using a connective tissue graft (CTG) is related to several aspects: the quantity of existing KTW [3], the thickness of the gingival mucosa, the dimensions of the recession, and the operator’s skills [4].

Although it is possible to use soft tissue from edentulous crests and gingival areas for grafts, the palatal mucosa is preferred due to its availability and ease of acquisition. Free gingival grafts (FGG) were the first to cover gingival recessions, increase the gingival mucosa thickness, and increase the keratinized tissue height [5]. They are taken from a superficial layer of the hard palate consisting mainly of lamina propria, containing a more significant amount of fibrous connective tissue and a lower percentage of adipose tissue [6]. On the other hand, the subepithelial CTG, taken from a deeper layer of the palate, consists mainly of submucosal tissue [6].

Several authors argue that soft tissues may vary in thickness between individuals and within the oral cavity, depending on several factors: race, age, genetic factors [7,8,9], bodyweight [10], periodontal phenotypes, arch size [11], and gender [12]. Bleeding and paresthesia are frequent complications after CTG harvesting [13]. So, it is essential to select a zone where an adequate amount of tissue can be obtained without causing important health risks [14]. The palate mucosa has been evaluated using different techniques, some more clinically invasive than others, to provide surgeons with more information. Measures using endodontic files [15], anesthesia needles [16], histological sectional sizes [6], and, mainly, periodontal probes [7,8,9,10,15] are considered more invasive. On the other hand, computed tomography (CT) [17], cone-beam computed tomography (CBCT) [18], and ultrasonic devices [11] are considered non-invasive; however, CBCT has the drawback of ionizing radiation.

Moreover, several CTG surgical techniques have been described. In 2009, Mcleod et al. [19] performed a de-epithelialized CTG, an FGG without the epithelial layer, removed from the hard palate before graft excision and explained that it provided greater control of the de-epithelialization process. In contrast, Zucchelli et al. (2010) [20] reported a preference for de-epithelialization outside the oral cavity, where they checked if the epithelial tissue was removed entirely under different incidences of light.

After PPS, besides evaluating the recession clinical result, the clinician should also control the soft tissue stability in the donor area. Del Pizzo et al. [21] evaluated the initial healing of the palate based on color, comparing three different techniques: FGG, “single incision,” and “trapdoor.” They concluded that the “single incision” technique allowed for a faster and more complete epithelialization within two weeks, while the other two methods required four weeks. Those authors hypothesized that the healing delay in the FGG and “trapdoor” techniques might be due to removing the epithelial layer of the palate mucosa [21] and vertical discharges. Moreover, Keskiner et al. [22] carried out a study on the FGG technique and suggested that the filling of the intervention site would be faster with at least 2 mm of residual tissue, while if there were less than 2 mm, the filling could take more than six months, mainly at the center point. This finding indicates that second-intention healing does not occur uniformly over the entire length, but is more advanced at the edges [23].

Within this context, this pilot study aimed to evaluate the healing dynamics process of the hard palate after FGG harvesting, in the short term (three months after surgeries), through a non-invasive technique utilizing digital imaging technology and tridimensional (3D) analysis software to perform the study of this technique. Furthermore, assessment of the results found to verify the existence of a relationship between gender or age with tissue loss. The clinical significance associated with this pilot study was to demonstrate how the digital approach could easily be used to the obtention of measures.

## 2. Materials and Methods

**Figure 1:**
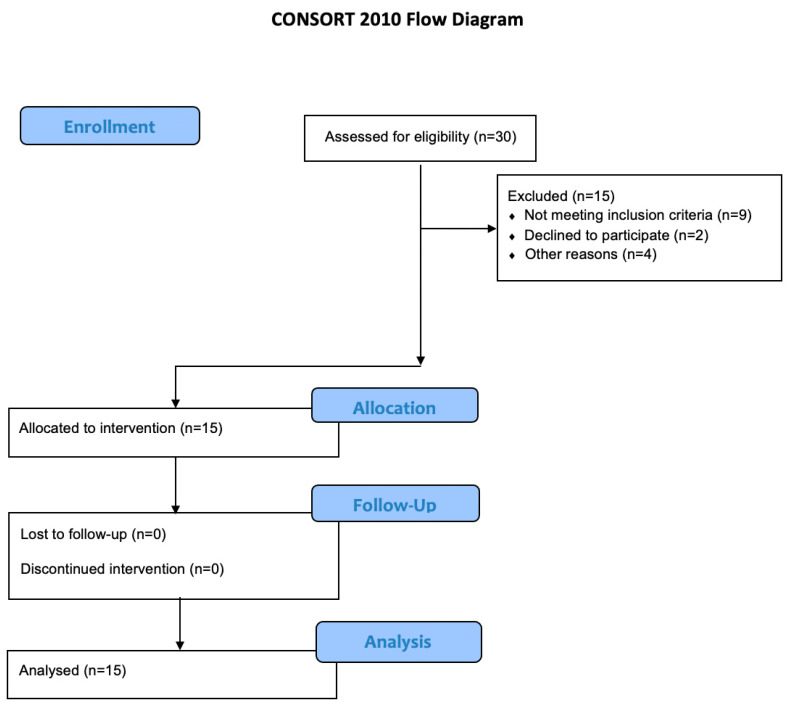
Consort flow diagram.

The study protocol was submitted and approved at the Ethical Committee of the Universidade Católica Portuguesa’s Ethics Committee (protocol number 25/2019—10.April.2019), designed in accordance with guidelines from CONSORT (http://www.consort-statement.org/), and in accordance with the Helsinki Declaration of 1975, as revised in 2013. Then, after selection (Figure 1), fifteen patients (*n* = 15) were included from the University Dental Clinic of the Faculty of Dental Medicine of the Universidade Católica Portuguesa (Viseu, Portugal) for a 3-month clinical evaluation.

### 2.1. Eligibility Criteria

The inclusion criteria were: good periodontal health (full-mouth plaque ≤20%, bleeding scores <10%, no pocket depths >3 mm, no active periodontal disease); no previous CTG; good systemic health (ASA classifications I and II); at least one buccal or lingual recession type 1 or 2 (RT1 or RT2) (Cairo’s classification) [24] gingival recession defect in upper and/or lower central and lateral incisors, canines, first and second premolars, and first molars; clinical indication (esthetic or sensibility) and/or patient request for recession coverage; and radiographic evidence of sufficient interdental bone (i.e., the distance between the crestal bone and the cementoenamel junction is not greater than 2 mm).

Patients with uncontrolled systemic diseases, pregnant or nursing, psychiatric problems, immunocompromised, or smoking ten or more cigarettes per day were excluded.

### 2.2. Patient Screening and Informed Consent

The patients received complete information about the periodontal treatment, all procedures, the aim of the study, and the need to attend follow-up appointments. Afterwards, the enrolled patients (Figure 1) signed the written informed consent before undergoing treatment. All personal data, including treatment-related medical information, were treated with absolute confidentiality by the study personnel. Each patient authorized the publication of the study results when they gave their informed consent to participate.

### 2.3. Surgeries and Connective Tissues Grafts Harvesting

All surgeries were performed by one expert surgeon (T.M.). Fifteen tissue grafts were harvested using the technique described by Zucchelli et al. [20]. A region of interest (ROI) was delineated for all patients (from the distal of the canine to the mesial half of the first molar) through two horizontal incisions, coronal performed 1.0–1.5 mm apical to the gingival margin. Two vertical incisions were traced to delimitate the area to be harvested. The blade was almost perpendicular to the bone plate along the horizontal coronal incision. Once an adequate soft-tissue thickness was obtained, it was rotated to become almost parallel to the superficial surface.

The graft’s thickness was maintained uniform while proceeding apically with the blade. Care was taken not to remove the periosteum, which protected the underlying bone and kept the graft’s size as uniform as possible. Once the graft was separated, the fatty tissue was eliminated and, sequentially, it was de-epithelized after being collected to be applied in the root coverage surgery. Patients were prescribed Ibuprofen 600 mg twice daily and chlorhexidine mouthwash 0.2% three times daily for two weeks.

### 2.4. Patient Evaluation and Data Collected

The assessment was performed in two periods: surgery day (T0) (one impression with alginate (Zhermack, Orthoprint Alginate): before the procedure) and another three months after surgery (T1). The sample included 15 unilateral palatal donor sites. The digital evaluation protocol was as follows: the patient’s casts were obtained at T0 before any anesthetic infiltration and T1 scanned by a Dental Wings^®^ scanner (Straumann). 3D digital analysis of the intervened areas was done with the software Geomagic Control X^®^ (v. X2021, Green Cove Springs, FL, USA) and Materialise Magics^®^ (v. 26, Plymouth, MI, USA). The US. The following variables were analyzed: mean thickness alterations ($\bar{x}$ TA), maximum thickness loss (MTL), mean maximum thickness loss ($\bar{x}$ MTL), and volume alterations (VA). A descriptive and bivariate analysis of the data was done.

Two trained clinicians (S.R. and T.M.) carried out all data collection. The data obtained were organized in the Excel^®^ program (Microsoft Corporation, Redmond, DC, USA) before proceeding to statistical analysis, using frequency tables, graphs, and statistical measures (mean, median, standard deviation, maximum and minimum, and frequencies).

Inferential analysis was performed using the IMB SPSS^®^ 25.0 program (Chicago, IL, USA). The Kolmogorov–Smirnov test was performed, with the correction of ‘Lilliefors,’ to assess if the variables were normally distributed. Non-parametric tests were then applied to paired samples to compare the two groups according to time and gender or age and compare distributions. The Mann–Whitney U test was used to verify if there were significant differences between T0 and T1 in ($\bar{x}$ TA), (MTL), ($\bar{x}$ MTL), (VA), and paired *t*-tests and Wilcoxon test were also used. The non-parametric Wilcoxon test was used when normality was not found. The level of statistical significance was set at 5% (*p* < 0.05).

### 2.5. Measurements Obtained by Software Analysis

ROI was kept during the entire study for each patient, which was verified in each impression, allowing the calculation of volume alterations. The STL files were superimposed in Geomagic^®^ software (v. X2021, Green Cove Springs, FL, USA) using the ‘best-fit algorithm’ for a precise evaluation during the study.

**Figure 2:**
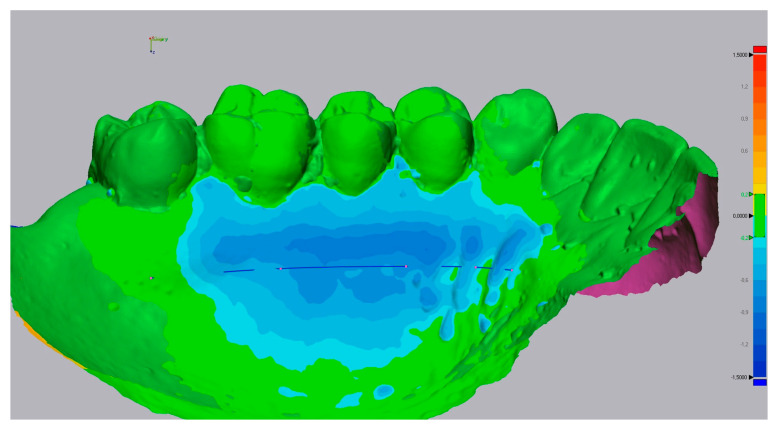
Insertion of a sagittal section line.

**Figure 3:**
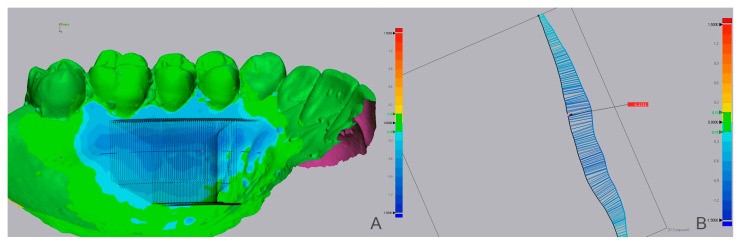
Case showing extensive tissue loss by the blue color. (**A**) Insertion of section plans perpendicular to ROI’s entire length. (**B**) Sagittal section and measurement of thickness loss points.

**Figure 4:**
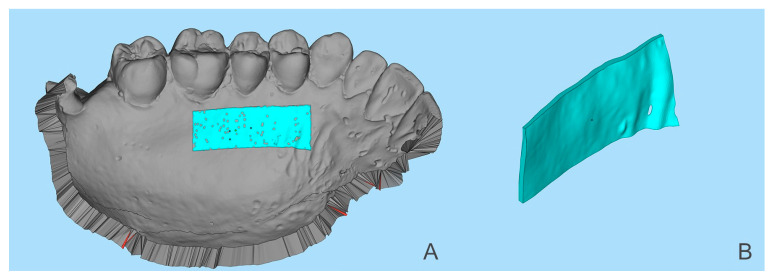
(**A**) Superimposed STL files of T0 and T1 with the region of interest (ROI) highlighted. (**B**) Region of interest with the volume alterations (VA) measured in mm^3^.

Then, using the ‘3D compare’ function, a 3D map was created showing the volumetric alterations in the palate (±0.15 mm tolerance, where green represents perfect alignment and blue shows loss of volume). Using still Geomagic^®^, a line was drawn to define a sagittal section of the STL models (Figure 2). Several perpendicular measurements were made along the line from the distal of the canine to the mesial half of the first molar, creating a standard rectangular section with measurements at every 0.3 mm (Figure 3). This method allows measuring the tissue’s thickness and determining the ‘point of maximum loss’, the ‘average of the points of maximum loss’ along the palate, and the ‘average thickness alteration’ (Figure 3A). The volume of tissue loss was calculated with the software Materialise Magics^®^ (v. 26, Plymouth, MI, USA). Volumetric changes were calculated in the ROI using Boolean operations to obtain the volumetric variation in the different models, in mm^3^ and relative percentage (Figure 4).

## 3. Results

Fifteen clinical cases were analyzed, 11 females (73.3%) and four males (26.7%). The patients’ average age was 28 ± 8.52 years (ranging between 16 and 48 years old). The patients’ age distribution did not follow the normality, which the many patients below the average age justified.

The measurements of each case for the post-operative period are provided in Table 1. Individually, variable values were mainly negative (indicating tissue loss), except in patients one and six. Mean values and other statistical measures were calculated for the variables $\bar{x}$ TA, $\bar{x}$ MTL, and VA, which generally compare both post-operative times. However, the mean values had few alterations between the post-operative evaluations at three months (T1), which showed an $\bar{x}$ TA of −0.26 mm (±0.31 mm), an $\bar{x}$ MTL of −0.81 mm (±0.49 mm), and a VA of 46.99 mm^3^ (±47.47 mm^3^). Nonetheless, each case was unique, following its healing pattern.

**Figure 5:**
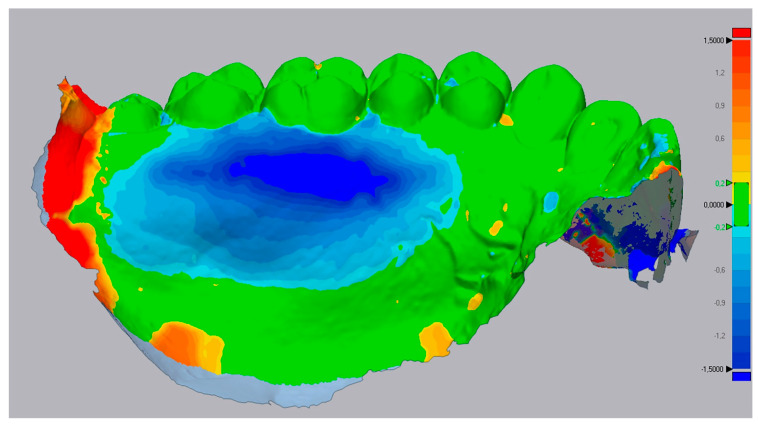
Case 7’s 3D evaluation shows extensive loss marked by the deep dark blue color.

All participants demonstrated tissue loss in the first three months post-surgery, except in case #1. The positive values (Table 1) showed a yellow-orange color spectrum, indicating tissue gain. No explanation for this event was found in the literature. One of the subjects stood out for a more significant loss (case #7) (Figure 5), with a $\bar{x}$ TA 1= −1.10 (±0.34 mm); the surgeon reported that the procedure was more invasive in harvest depth, which might have contributed to this follow-up.

In several cases, tissue loss was commonly seen in the palatine rugae area, easily distorted by soft tissue compression during alginate impressions. Furthermore, the arch’s width may influence the scan’s accuracy and cause changes in the color map below the ROI and far from the surgical area.

Individuals aged <28 years old (*n* = 9) and ≥28 years (*n* = 6) were subjected to a comparative analysis. No significant differences were found (*p* > 0.05); thus, there was no relationship between age and any variables analyzed (Table 2). The genders were also subjected to a comparative analysis, and, equally, no significant differences were found (*p* > 0.05); thus, there was no relationship between gender and any variables analyzed (Table 3).

## 4. Discussion

In periodontics, cast model digitization and 3D analysis have been described to study the healing dynamics and root coverage success, using the CEJ line as a reference [25]. There are other methods for carrying out similar studies, such as ultrasonic devices [26] and CBCT, but this involves an exposure to radiation, a lower cost, and a greater comfort to the patient [18]. In the present pilot study, none of these methods were applied, respectively, due to not having the ultrasonic device available and to avoid the patients being exposed to radiation twice (T0 and T1) in a short period. In addition, the literature suggests that CBCT is not the most suitable method for measuring soft tissues [27].

To the best of our knowledge, our group was the first to report in the literature, in 2019, the 3D evaluation of the healing process from the palate donor site [28]; and in this study, a 3D digital measuring method was used for the first time in a clinical trial to evaluate the outcomes after surgical graft harvesting from the hard palate. This recently developed innovative method was initially described to measure in vitro alveolar ridge defects [29]. It can now be used to record soft tissue contour and volumetric changes in several clinical scenarios.

Numerous methods are described in the literature for measuring soft tissue thickness. Using a needle may pierce the periosteum or the palatal bone, inducing errors with higher values [17], so using the periodontal probe is preferred [13]. Another probing method uses an endodontic file with a silicone stop, but its displacement can influence the measurements [30]. Moreover, all these methods require a reproducibility guide and anesthesia and are subject to operator error, being more appropriate for surgical procedures than pre- and post-surgical evaluations [31]. Bypassing the pain issue, the ultrasound was suggested as a non-traumatic alternative and proved faster and more accurate than the other methods [26]; however, it is a sensitive device requiring multiple measurements to overcome possible errors [11]. Even so, very few investigations have actually attempted to measure or quantify soft tissue thickness in the graft harvesting region.

None of the previously described methods were established as a standard for the dimensional analysis of soft tissues. Hence, the scanning may be considered a promising method, as it allows using digital software to measure the thickness at various points and volumes, defining the areas to be analyzed. This digital method offers significant advantages, including its non-invasive nature, a high reproducibility, and an excellent measurement accuracy. It provides an unforeseen precision in evaluating surgical graft harvesting regarding both two-dimensional measurements and 3D evaluations (soft tissue thickness/volume). Anyhow, these measurements require training and time.

The 3D changes caused by the CTG removal can be visually assessed, represented in a color map where cold colors indicate zones of volumetric loss, warm colors represent zones of volumetric gain, and a green color indicates a perfect overlay of the STL files. However, the variables’ calculations must be discussed for each clinical case due to the existing variability. In this study, the variabilities were observed to exclude possible errors related to the anatomical diversity of the palate and teeth size, obtaining a recovery percentage or additional loss.

Special attention was given to the subjects of cases #3 and #13, who were smokers (<10 cigarettes/day). Where case #3 showed a minor thickness loss (−0.04 ± 0.10 mm), case #13 showed a greater loss (−0.23 ± 0.30 mm) (Table 1). Smokers usually have a delay in the epithelialization of the palate. Silva et al. (2010) [32] found that, after two weeks, 92% of non-smokers appeared to have complete epithelialization compared to 20% of smokers. Therefore, our result showed no influence of the smoke on the palate healing.

Concerning age and gender, studies suggest that aging comes with a decrease in levels of growth factors. Processes of re-epithelialization, collagen synthesis, angiogenesis, and wound contraction are delayed. These factors could contribute to variations in the results of the cases described. Moreover, estrogen and androgens seem to influence the healing mechanisms, being slower in males than in females. However, the collected data did not show a relationship between age or gender and 3D tissue healing. A larger sample with a broader age range would probably provide more reliable results.

The measuring technique applied in this study has some limitations. As measurements were based on evaluating soft tissue contour changes over time, it is impossible to quantify the thickness of the soft tissue harvested accurately. In that sense, an intraoperative intervention (mold) could be done to calculate the thickness of harvested tissues. Moreover, the scan could be performed directly intraoral, facilitating the daily routine, even though it is not free from errors during scanning. According to the manufacturer’s specifications, in full-arcade scanning, the error associated with the scanner’s image capture is 50 μm; however, it has been described that this error could reach around 100 μm.

Furthermore, it is suggested to increase the sample size once 15 patients are a low number. Therefore, a recent study published by Tavelli et al. (2022) [33] used 19 patients applying a similar technique. In addition, three months is a short period of analysis, which is recommended to extend to study after 6 and 12 months in order to obtain a robust analysis of the healing dynamics per case. Nonetheless, authors [33] tested all those periods and affirmed that three months is the best analysis period once the donor site undergoes the greatest volumetric changes in the first three months.

Furthermore, the lack of information regarding the graft thickness obtained must be standardized, as carried out by Keskiner et al. [22]. These authors reported a relationship between residual thickness and healing efficiency and detected differences between the edges and the central points of the donor area. In fact, most cases show variations regarding volumetric recovery, with some “islands” of loss with light blue edges and darkened central points representing more pronounced losses. Another feature noticed is tissue neoformation, which often leads to scar formation [23], visible in the STL images in most cases.

Within the limitations of this pilot study, CTG harvesting promoted palatal soft tissue changes, with standard volume and thickness loss. The soft tissue healing process demonstrated continuity throughout the 3-month follow-up. The 3D digital evaluation was a non-invasive method, allowing the study of soft tissue healing dynamics, a reproducible technique for measuring thickness or volume after connective tissue is collected. There was no relationship between age/gender and any variables analyzed, even though the numbers were greater in older patients. More robust studies should be developed to verify the influence in the more significant healing period.

## Figures and Tables

**Table 1:** Variable values obtained by T0-T1 STL intersection.

Case Number	$\bar{x}$TA (mm)	MTL (mm)	$\bar{x}$MTL (mm)	VA (mm^3^)
**1**	0.18 ± 0.12	−0.23	0.08 ± 0.06	4.18
**2**	−0.15 ± 0.11	−0.66	−0.27 ± 0.04	30.90
**3**	−0.04 ± 0.10	−0.45	−0.23 ± 0.03	2.72
**4**	−0.03 ± 0.12	−0.47	−0.15 ± 0.07	3.47
**5**	−0.19 ± 0.17	−1.08	−0.47 ± 0.04	37.20
**6**	0.04 ± 0.08	−0.20	−0.03 ± 0.06	2.19
**7**	−1.10 ± 0.34	−2.15	−1.49 ± 0.14	161.61
**8**	−0.58 ± 0.13	−0.91	−0.68 ± 0.09	90.90
**9**	−0.30 ± 0.13	−0.70	−0.39 ± 0.06	52.48
**10**	−0.61 ± 0.24	−1.23	−0.85 ± 0.10	131.52
**11**	−0.16 ± 0.11	−0.46	−0.27 ± 0.04	16.92
**12**	−0.21 ± 0.18	−1.31	−0.62 ± 0.04	35.54
**13**	−0.23 ± 0.30	−0.81	−0.44 ± 0.06	56.74
**14**	−0.25 ± 0.13	−0.79	−0.41 ± 0.05	41.51
**15**	−0.26 ± 0.16	−0.75	−0.40 ± 0.07	37.07

VA = Volume alterations; $\bar{x}$ MTL = mean of maximum thickness loss; MTL = Maximum thickness loss; $\bar{x}$ TA = mean thickness alteration.

**Table 2:** Age comparative analysis after three months.

Age								
Variables	<28 Year			≥28 Year			Mann–Whitney U	
	Mean	SD	*n*	Mean	SD	*n*	U	*p*-Value
**MTA (mm)**	−0.19	0.19	9	−0.37	0.43	6	18.000	**0.289**
**MTL (mm)**	−0.70	0.37	9	−0.98	0.64	6	18.000	**0.289**
**VA (mm^3^)**	36.94	41.10	9	62.08	56.14	6	16.000	**0.195**

SD = Standard Deviation; VA = Volume alterations; MTA = Maximum thickness alteration; MTL = Maximum thickness loss.

**Table 3:** Gender comparative analysis after three months.

Gender								
Variables	Female			Male			Mann–Whitney U	
	Mean	SD	*n*	Mean	SD	*n*	U	*p*-Value
**MTA (mm)**	−0.26	0.18	11	−0.25	0.58	4	13.000	**0.240**
**MTL (mm)**	−0.81	0.32	11	−0.83	0.89	4	15.000	**0.361**
**VA (mm^3^)**	48.45	35.64	11	42.99	79.08	4	14.000	**0.296**

SD = Standard Deviation; VA = Volume alterations; MTA = Maximum thickness alteration; MTL = Maximum thickness loss.
